# A spatial assessment of mercury content in the European Union topsoil

**DOI:** 10.1016/j.scitotenv.2020.144755

**Published:** 2021-05-15

**Authors:** Cristiano Ballabio, Martin Jiskra, Stefan Osterwalder, Pasquale Borrelli, Luca Montanarella, Panos Panagos

**Affiliations:** a European Commission, Joint Research Centre (JRC), Ispra, Italy; b Environmental Geosciences, University of Basel, Basel, Switzerland; c Université Grenoble Alpes, CNRS, IRD, Grenoble INP, IGE, Grenoble, France; d Università degli Studi di Pavia, Dipartimento di Scienze della Terra e dell'Ambiente, Pavia, Italy

**Keywords:** Soil contamination, Mercury, Hg, Mining, Coal, Deep neural networks

## Abstract

Mapping of surface soil Hg concentrations, a priority pollutant, at continental scale is important in order to identify hotspots of soil Hg distribution (e.g. mining or industrial pollution) and identify factors that influence soil Hg concentrations (e.g. climate, soil properties, vegetation). Here we present soil Hg concentrations from the LUCAS topsoil (0–20 cm) survey including 21,591 samples from 26 European Union countries (one sample every ~200 km^2^). Deep Neural Network (DNN) learning models were used to map the European soil Hg distribution. DNN estimated a median Hg concentration of 38.3 μg kg^−1^ (2.6 to 84.7 μg kg^−1^) excluding contaminated sites. At continental scale, we found that soil Hg concentrations increased with latitude from south to north and with altitude. A GLMM revealed a correlation (R^2^ = 0.35) of soil Hg concentrations with vegetation activity, normalized difference vegetation index (NDVI), and soil organic carbon content. This observation corroborates the importance of atmospheric Hg^0^ uptake by plants and the build-up of the soil Hg pool by litterfall over continental scales. The correlation of Hg concentrations with NDVI was amplified by higher soil organic matter content, known to stabilize Hg in soils through thiol bonds. We find a statistically significant relation between soil Hg levels and coal use in large power plants, proving that emissions from power plants are associated with higher mercury deposition in their proximity. In total 209 hotspots were identified, defined as the top percentile in Hg concentration (>422 μg kg^−1^). 87 sites (42% of all hotspots) were associated with known mining areas. The sources of the other hotspots could not be identified and may relate to unmined geogenic Hg or industrial pollution. The mapping effort in the framework of LUCAS can serve as a starting point to guide local and regional authorities in identifying Hg contamination hotspots in soils.

## Highlights

• The first high resolution assessment of mercury in European Union topsoil
• Median Hg is estimated to 38.3 μg·kg^−1^ with 10% exceeding the 84.7 μg·kg^−1^.
• Topsoil pool of Hg in EU is estimated to c.a 44.8 Gg.
• Hg content is correlated with organic matter, temperature, NDVI and landcover.
• 209 hotspots were identified with concentrations >422 μg kg^−1^, 42% related to mining.

## Graphical abstract

**Figure:**
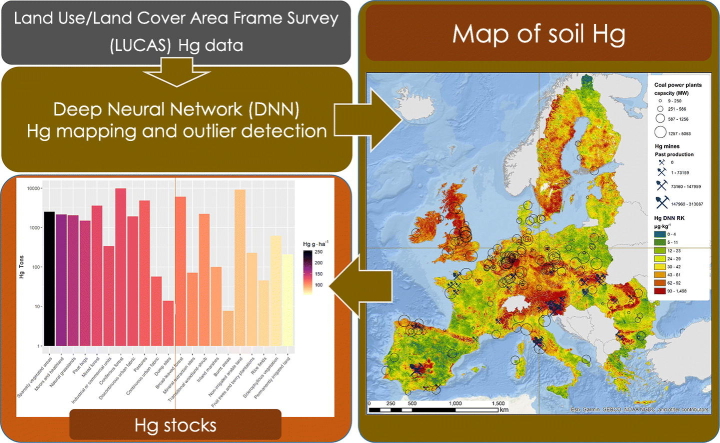

## 1 Introduction

Soils are a major sink for metals released into the environment by both natural processes and anthropogenic activities. Soil contamination especially with toxic elements such as arsenic (As), lead (Pb) and mercury (Hg) poses a significant risk to human health (Järup, 2003). Hg is ranked third by the US Government Agency for Toxic Substances and Disease Registry due to its abundance, toxicity and potential for human exposure (ATSDR, 1999). Owning to its long-range atmospheric transport and its bioaccumulation properties, mercury has officially been recognised as a global pollutant by the 2013 UN's legally binding Minamata Convention (United Nations, 2013). The Minamata convention on mercury aims to limit the significant health and environmental impacts of Hg pollution by reducing anthropogenic emissions to the atmosphere and release into soils and surface waters. The convention also addresses provisions for mining, import/export and waste management of products containing Hg (United Nations, 2013). Policy actions at national, regional, and global scales have also addressed Hg pollution sources. In the United States, the recent Hg and Air Toxics Standards mandate Hg emissions reductions for the first time from power generation sources, in particular, coal-fired power plants. Experience with regional Hg management suggests that future policy should take into account transboundary influences, coordinate across environmental media, and better assess human and ecological impacts in regulatory analyses (Lamborg et al., 2014).

On a global scale, current anthropogenic emissions add approximately 2500 Mg Hg to the atmospheric Hg pool each year. The uncertainty of the emission flux ranges between 2000 and 2820 Mg A^−1^. Natural sources such as weathering of Hg-containing rocks, geo-thermal activities and volcanic eruptions sum up to about 500 Mg Hg A^−1^ (UNEP, 2019). The main Hg emission sources to air in Europe originate from stationary combustion (mainly burning of coal) and the non-ferrous metal industry (UNEP, 2019). Anthropogenic Hg emissions to the atmosphere resulted in an overall ~300% increase in Hg deposition to the terrestrial environment compared to natural levels (defined as pre-1450 CE levels). This resulted in an increase of the overall Hg concentration in surface organic soils by about 15% (Obrist et al., 2016; Outridge et al., 2018). Total anthropogenic releases and emissions of Hg have cumulated to 1540 (1060–2800) Gg (10^9^ g), of which 470 Gg were emitted directly to the atmosphere and 1070 Gg were released to soils and surface waters (Streets et al., 2017). To understand the fate of Hg in soils, it is essential to quantify Hg storage in soils and model the processes that led to the build-up of the Hg soil pool and control Hg release to the aquatic ecosystems and emission to the atmosphere.

Generally, more than 80% of Hg in surface soils originates from atmospheric inputs via wet and dry deposition, directly onto soils or indirectly via plant surfaces with throughfall (Demers et al., 2013; Jiskra et al., 2015; Obrist et al., 2017; Zheng et al., 2016). In soils, Hg is strongly bound to soil organic matter (in particular thiols), clay minerals and Fe and Al oxides (Meili, 1991; Skyllberg et al., 2006; Yin et al., 1996). Several studies assessed the distribution of Hg at continental scales. The U.S. Geological Survey analysed soil Hg concentrations from 1911 randomly distributed samples in the United States and found average soil Hg concentrations of 24 μg kg^−1^ in topsoil (A-horizon, typically 0–20 cm) and 22 μg kg^−1^ in the parent material (C-horizon) (Smith et al., 2013). Only 2.6% of data exceeded 100 μg kg^−1^. Obrist et al. (2016) reported that soil Hg distribution in the western United States was strongly associated with precipitation, soil organic carbon, canopy greenness and mass of Hg in foliage. The authors concluded that biomass production controlled by water availability was an important factor for leaf uptake of atmospheric Hg and Hg accumulation in soils. As a result, highest soil Hg levels were found in productive landscapes and low soil Hg concentrations in barren and shrubland soils (Obrist et al., 2016). This emphasizes the importance of gaseous elemental mercury (Hg^0^) uptake by vegetation for soil Hg inputs via litterfall (Jiskra et al., 2018; Wang et al., 2016). A soil survey in China, covering 3382 sampling sites reported a median Hg concentration of 26 μg kg^−1^ (13 to 56 μg kg^−1^, interquartile range) in surface soils (0 to 25 cm) with ≈7% of all soil data exceeding 150 μg kg^−1^(Wang et al., 2015).

In Europe, Lado et al. (2008) modelled the spatial distribution of Hg in topsoil in 28 countries using 1588 soil samples from the Forum of European Geological Surveys (FOREGS) geochemical database (Salminen et al., 2005). According to their findings, the median Hg value in soils for Europe was about 40 μg kg^−1^. Northern European soils typically contain more Hg in comparison with Central and Southern European soils because a wet and cold climate favours the build-up of soil organic material (Ottesen et al., 2013). The project Geochemical Mapping of Agricultural and Grazing Land Soil in Europe (GEMAS), that was based on 2211 samples from agricultural land (0–20 cm; median of 30 μg kg^−1^) and 2118 samples from permanent grasslands (0–10 cm; median of 35 μg kg^−1^) identified high Hg concentrations close to urban centers such as Paris and London (Reimann et al., 2018a).

High local soil Hg concentrations, might be due to either high geogenic Hg levels, anthropogenic pollution through mining (Gosar et al., 2016; Millán et al., 2006), operation of base-metal smelters (Eckley et al., 2015; Eckley et al., 2011) or chemical manufacturing processes (Osterwalder et al., 2019; Zhu et al., 2018). Most important Hg mining areas in Europe include the Idrija mine in Slovenia (Gosar et al., 2016) and the Almadén mine in Spain (Millán et al., 2006) where high geogenic Hg levels occur and soil Hg concentrations exceed 10^6^ μg kg^−1^. Centuries of mining activities have contributed to a dispersion of Hg contamination in soils through mine tailings, enhanced water and wind erosion of outcrops and processed ore storages. Elevated Hg concentrations in soils contaminated through airborne transport of Hg have been reported in proximity to point sources (smelting of metals, e.g. Hg, Pb (Ettler et al., 2007), and Zn (Estrade et al., 2011)) and coal combustion (Dragović et al., 2013). The vicinity of metal industry or coal-fired power plants especially in the border area of the Czech Republic, Germany and Poland was an important driver in Hg spatial distribution (Reimann et al., 2018a).

According to literature, there are two main factors controlling Hg distribution in soils on a local to continental scale: a) naturally enriched Hg sites such as geothermal systems and proximity to related anthropogenic activities such as mining and ore processing; b) vegetation activity and carbon build-up in soils that control the accumulation of atmospheric Hg in soils.

The objective of this study was to use the 21,591 soil Hg concentration data from the Land Use/Land Cover Area Frame Survey (LUCAS) in order to assess the topsoil Hg distribution across the European Union. We aim to: 1) calculate the topsoil Hg pool and identify the spatial distribution of topsoil Hg concentrations for 26 member states of the European Union, 2) test the hypothesis whether more productive landscapes with high vegetation greenness (NDVI) and large soil organic carbon concentrations show elevated soil Hg concentrations, and 3) identify hotspots of Hg contamination due to naturally occurring Hg deposits, mining, and industrial use of Hg.

Given the sample size of the LUCAS survey and its extensive spatial coverage, this study provides an improved map, with a high spatial resolution (250 m) of the distribution of Hg in the topsoil of EU. Moreover, LUCAS provides information not limited to agricultural soils, but covers a broad range different soil, parent material and land cover types, this together with the huge number of samples allows to better model the interplay of factors influencing Hg distribution and provides more reliable estimates of Hg stocks in EU.

## 2 Methods and data inputs

### 2.1 Sampling and laboratory analysis of topsoil samples

Topsoil characteristics across all Member States of the European Union (EU) are regularly acquired through the Land Use/Land Cover Area Frame Survey (LUCAS), which is a regular harmonized survey, performed every three years to gather information on land cover and land use. Since 2009, the survey also includes operations of sampling and analysing of the main properties of EU topsoil. A thoughtful description the LUCAS survey organization in given in Orgiazzi et al., 2018.

The LUCAS topsoil surveys are performed collecting one soil sample every ~200 km^2^ (Panagos et al., 2013). It corresponds to 21,682 samples collected from EU member states in 2009. The sampling operation consist in the collection of five topsoil samples (0–20 cm) for each LUCAS location. These subsamples are mixed together, air-dried and transferred to a single central laboratory for the analytical procedures. The analytical procedures include the acquisition of both physical and chemical soil properties.

In addition to the standard chemical and physical analyses, LUCAS 2009 topsoil samples were analysed to quantify heavy metals, cadmium (Cd), cobalt (Co), chromium (Cr), copper (Cu), iron (Fe), mercury (Hg), manganese (Mn), nickel (Ni), lead (Pb), antimony (Sb) and zinc (Zn). Additionally, magnesium (Mg) and arsenic (As) content were measured. The heavy metals content of the 21,682 soil samples was measured applying the ISO 11466: 1995 method using aqua regia as the extracting agent. The sample treated by microwave assisted digestion (Nemati et al., 2010) were then processed for metal analysis by ICP-OES (Inductively Coupled Plasma – Optical Emission Spectrometry) (Cristache et al., 2014). Additionally, a subset of 500 randomly selected LUCAS samples were also analysed with two further procedures employing the open vessel digestion and the microwave assisted digestion. This cross-comparison procedure confirmed a good general agreement between the two approaches (Cristache et al., 2014).

The detection limit for topsoil Hg analysis was 0.054 μg kg^−1^ and only 30 samples were below this threshold. For those samples, we have assigned the value of 0.027 μg kg^−1^ as a mean between zero and the lower detection limit. The quality of the analysis was confirmed by assays on reference material and repeated analyses of randomly selected samples (Panagos et al., 2018).

### 2.2 Deep learning model for topsoil Hg distribution

The spatial distribution of mercury in soils depends on a series of variables, both natural and anthropic, influencing the emission, transport and deposition of Hg in the environment. As mercury transport is mostly due to atmospheric processes, modelling the spatial distribution of this element involves accounting for multiple interconnected factors acting on its transport. For instance, distance from point sources is a critical covariate, however considering just distance is often useless if other parameters, such as wind intensity and direction, are not taken into account. Nevertheless, using a high number of variables for spatial modelling poses several problems related to data collinearity and dimensionality. Following with the example of wind influence, wind direction and intensity are locally correlated (wind coming from a certain direction are likely to have a given intensity in a specific area and at a specific time), hence the issue of collinearity. Moreover, much of the covariates that can be used for mapping are unstructured data, like raw data from remote sensing images of atmospheric data. For instance, we do not have access to precise topsoil temperature measurements, but we can use proxy data derived from ground radiance. However, these proxies are not directly translatable into the desired measures, due to the interplay of other factors (i.e. soil humidity, soil cover, etc.).

In order to model soil Hg distribution, a Deep Neural Network (DNN) feed forward model was applied. DNN are models especially apt at extracting useful information from unstructured data (Adnan and Akbar, 2019). In a feed forward DNN model, data flows from the input to the output layer without looping back. The advantage of DNNs is their capacity to model non-linear relationships by generating layered compositions of the input. Compared to shallow networks (i.e. composed of a single layer), the additional layers of a DNN allow for complex combinations of features derived from the lower layers. This makes possible for DNN to model very complex data with, relatively, fewer units than shallow network. Recently, DNN have been successfully applied for mapping different soil properties (Behrens et al., 2018; Padarian et al., 2019).

**Fig. 1:**
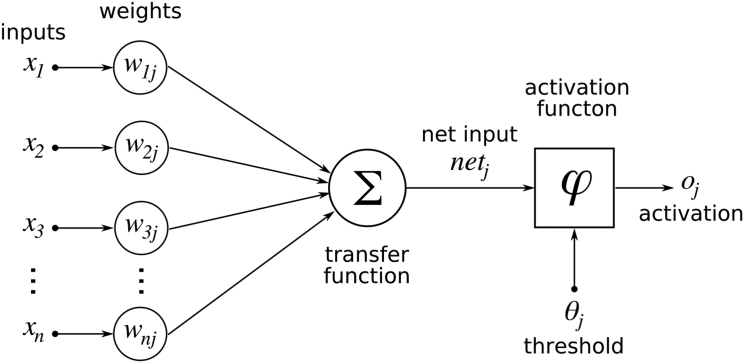
Representation of an artificial neuron.

As in common neural networks, the DNN is built by creating a map of *n* virtual neurons based upon the inputs *x*, and by assigning random numerical weights (*w*) their interconnections (Fig. 1). The virtual neuron in the model is analogous to a biological neuron, where the neurons' output is transmitted along synapses and it is then aggregated as input for the next connected neuron. In the virtual neuron, the input signals are aggregated as weighted combination

(1) $$\varphi \left(\alpha \right)=\sum_{i=1}^{n}w_{i}x_{i}+b$$

where *α* is the aggregated signal, *w*_*i*, …, *n*_ are the weights, *x*_*i*, …, *n*_ represent the inputs and *b* is the bias. The φ(*α*) represents a nonlinear activation function by the network, while the bias *b* is the neuron's activation threshold. The network is trained by adjusting the weights so that its output matches as possible the dependent variable. Given the potentially large number of layers, resulting in the capacity of approximating very complex functions, DNNs are prone to overfitting. This occurs as the DNN will approximate not only the target variable signal, but also its noise. Regularization, weight decay or sparsity regularization can be applied during training to reduce overfitting.

The network applied in this study consists of four hidden layers with 2000, 1000, 500 and 200 neurons. The activation function is a rectifier with dropout function and the network adopts L1 and L2 regularization. L1 regularization adopts a complexity penalty to the absolute value of the weights in the loss function, whereas in L2 the complexity penalty is used on the square of the weights. In both cases the regularization shrinks the values of weights so that the network retains only the most informative features. Regularization is particularly effective in models with a large number of features where other methods, as cross-validation or stepwise selection, would be inefficient.

### 2.3 Auxiliary variables

A series of variables, potentially influencing the topsoil Hg distribution were considered in this study. The variables can be grouped as follows: land cover, natural and anthropogenic sources of Hg (Table 1) owning to their influence on Hg distribution. As a compromise between the spatial resolution of the Moderate Resolution Imaging Spectroradiometer (MODIS) data (500 m), the resolution of the digital elevation model (DEM) (25 m) (Bashfield and Keim, 2011) and the resolution of the WorldClim climatic datasets (1000 m) (Fick and Hijmans, 2017), a spatial resolution of 250 m was chosen for this study.

**Table 1:** Spatially continuous covariates used in the deep learning model for topsoil Hg distribution on a European scale.

Feature group	Environmental feature	Covariate	Source	#layers/classes	Covariate type
Land cover	Land cover	CORINE land cover type	CORINE	44	Categorical
Natural	Climate	Seasonal rainfall and air temperatures	Worldclim	12	Numerical
		Surface temperature	Landsat	1	Numerical
	Soil properties	Soil parent material	European Soil Database	50	Categorical
		Soil chemical and physical parameters	LUCAS	8	Numerical
	Topography	DEM derived topographic features	SRTM/EU DEM	13	Numerical
	Vegetation	EVI, MODIS reflectance	MODIS		Numerical
Sources of Hg	Coal power plants	Coal power plants density	Enipedia	1	Numerical
	Global Hg emissions	Global Hg emissions into atmosphere	AMAP/UNEP	1	Numerical
	Mining activity	Distance to Hg and Au/Ag mines	Mines4EU	3	Numerical

**Table 2:** LUCAS land cover types and land cover codes. The land cover derives from direct field observations. Bare land classes are grouped into a single class given their numerical scarcity.

Land cover	Detailed land cover type	Code
Artificial	Built-up areas	A1
	Artificial non-built areas	A2
	Other artificial areas	A3
Cropland	Cereals	B1
	Root crops	B2
	Non-permanent crops	B3
	Pulses, vegetables and flowers	B4
	Fodder crops	B5
	Fruit trees	B7
	Other permanent crops	B8
Woodland	Broadleaved forest	C1
	Coniferous forest	C2
	Mixed woodland	C3
Shrubland	Shrubland with sparse tree cover	D1
	Shrubland without sparse tree cover	D2
Grassland	Grassland with sparse tree cover	E1
	Grassland without sparse tree cover	E2
	Spontaneously re-vegetated surfaces	E3
Bare land	Bare land	F0
Waters	Inland water bodies	G1
	Inland running water	G2
Wetland	Inland wetlands	H1

#### 2.3.1 Climate

Monthly temperature and monthly precipitation estimates (minimum, maximum and mean values), along with bioclimatic variables were obtained from the WorldClim (http://www.worldclim.org/) dataset at a spatial resolution of 1000 m. WorldClim layers are the result of the interpolation of average monthly climate data collected from numerous weather stations. The interpolation is based on a thin plate smoothing spline using latitude, longitude and elevation as independent variables to locally interpolate clmatic data (Hijmans et al., 2005). The climatic variables used in the model included monthly averages of minimum and maximum temperatures and monthly rainfall rates, along with bioclimatic variables (temperature and precipitation indexes). Due to the presence of a strong collinearity among climate layers, a feature selection procedure was applied in the model training phase.

High resolution measured surface temperature was extracted from the Landsat (Data available from the U.S. Geological Survey) thermal band by merging several years in order to limit the influence of extreme climatic conditions. For the analysis, several thresholds of the thermal band were considered, with the most significant was found to be the 0.75 quantile of the yearly surface temperature.

#### 2.3.2 Parent material and soil properties

The covariate soil properties in the DNN was derived from the European Soil Database (ESDB) (Panagos et al., 2012). The ESDB was used as a multinomial variable identifying and labelling similar parent material types. See Table S1 in the Supplement for details on the parent material type and code.

Soil chemical and physical properties were included using the interpolated LUCAS data (Ballabio et al., 2019; Ballabio et al., 2016).

#### 2.3.3 Topography

European-scale terrain features were derived from the EU-DEM digital elevation model (EU-DEM, 2017). The elaboration of topographic features was done using the SAGA GIS software; features were then re-scaled to 250 m. Among the various terrain indices tested for their informative content, the most informative covariates were the Multi-resolution Valley Bottom Flatness (MRVBF) and the Multi-resolution Ridge Top Flatness (MRRTF) (Gallant and Dowling, 2003), along with elevation, slope, slope height and vertical distance to channel network (CNBL).

#### 2.3.4 Land cover

The CORINE (CORdinate INformation on the Environment) is a land cover spatial database, comprising 44 classes, derived from computer aided photointerpretation. The nominal scale of CORINE is 1:100,000 with a minimum mapping unit (MMU) of 25 ha and a change detection threshold of 5 ha. The reliability of the CORINE at 95% confidence level is 87.0 ± 0.7%, according to LUCAS data validation (Büttner, 2014). The CORINE 2012 dataset was used to represent the spatial distribution of land cover types and their influence on Hg distribution.

#### 2.3.5 Vegetation

In order to model vegetation dynamics and vegetation cover characteristics, MODIS Global Vegetation Indices (Didan, 2005) products for 2009 were used. These products are characterized by a spatial resolution of 250 m and a temporal resolution of 16 days. The products include blue, red and near- and mid-infrared reflectance, centered at 469 nm, 645 nm, and 858 nm, respectively. Reflectances are used to determine MODIS daily vegetation indices, such as the Normalized Difference Vegetation Index (NDVI) and the Enhanced Vegetation Index (EVI). NDVI is defined as

(2) $$\text{NDVI}=\frac{\mathrm{NIR}-\mathrm{RED}}{\mathrm{NIR}+\mathrm{RED}}$$

where NIR and RED are the reflectances measured in the near-infrared and visible (red) regions, respectively.

The EVI index is calculated as follows:

(3) $$\mathrm{EVI}=g∙\frac{\mathrm{NIR}-\mathrm{RED}}{\mathrm{NIR}+c1∙\mathrm{RED}-c2∙\text{BLUE}+L}$$

where *g* is a gain factor, NIR, RED, and BLUE are the respective regions surface reflectances, *L* is the canopy background adjustment, and *c*1 and *c*2 are coefficients for the aerosol resistance, which uses the blue band to correct for aerosol influences on the red band. The coefficients adopted in this study are; *L* = 1, *c*1 = 6, *c*2 = 7.5, and *g* = 2.5.

Vegetation dynamic was modelled from MODIS data using a first order harmonic model time series of the EVI and NDVI 16 days data. The harmonic model is based upon a discrete Fourier decomposition of temporal curves in a linear trend plus amplitude, variance and phase terms. The harmonic model can be defined as

(4) $$\hat{Y_{t}}=\alpha_{0}+\sum_{j=1}^{m}\alpha_{j}\;\cos\left(\frac{j2\mathrm{\pi t}}{l}\right)+\beta_{j}\sin\left(\frac{j2\mathrm{\pi t}}{l}\right)$$

where ${\hat{Y}}_{t}$ is the vegetation index value, *t* is the time value for a given pixel, *m* is the order of the polynomial (set as one in this study), *α*_*j*_ and *β*_*j*_ are the Fourier coefficients and *l* is the cycle length. Harmonic models of vegetation dynamics from Fourier series based on satellite data, have been used to model changes in the vegetation cover over time for several decades (Menenti et al., 1993; Moody and Johnson, 2001; Olsson and Eklundh, 1994) providing more explanatory and detailed spatial information on the different types of vegetation cover, than using composite images alone.

Additionally, a Principal Component Analysis (PCA) of the full MODIS 16 day images time series was performed for each band in order to extract relevant features. The PCA projects the time correlated data from MODIS into orthogonal PCA components ordered according to the percentage of data variance explained. Thus, the result is a series of maps where the first few PCA components account for most of the time related variation in each MODIS band.

#### 2.3.6 Anthropogenic sources of soil Hg contamination

The main sources of Hg contamination in European soils are 1) present and past mining activities, 2) legacy industrial contamination and 3) combustion of coal. In order to include the effect of mining activities, information on the geographical location, type of mineral extracted and past production of mines, were extracted from the Promine (http://promine.gtk.fi/) (Cassard et al., 2015) and the Minerals4EU (http://www.minerals4eu.eu/) (Lopes et al., 2018) databases. The distance from the mines was mapped as a continuous variable.

Mercury contamination from legacy industrial activities and atmospheric sources of Hg were based on the AMAP/UNEP dataset of Hg emissions (AMAP/UNEP, 2013). The dataset was interpolated at the resolution of the other covariates using ordinary kriging. However, given its coarse spatial resolution, the information it provided in the context of this study was limited.

Coal combustion for power production is another main source of Hg (Mukherjee et al., 2008; Yudovich and Ketris, 2005) as up to 90% of the Hg contained in the coal is released in the atmosphere. Modelling of coal emission was based on the data extracted from the Enipedia database (https://old.datahub.io/dataset/enipedia) (Davis et al., 2015) by deriving the spatial density of brown coal and hard coal firing power plants as a continuous layer.

### 2.4 Partial dependency plots and mixed models as global surrogates

DNN are a powerful approach for modelling unstructured data and are highly efficient in mapping Hg spatial distribution. However, DNN models, being based upon thousands of weights are not human interpretable and can be defined as a black-box. To avoid this issue, it is possible to create Partial Dependency Plots (also known as marginal effects plots in the regression context), where the influence of a given variable on the outcome is plotted. The PDP shows the change of the target variable average due following the variation of one covariate. The visualization of PDP allows to get the idea of how covariates and outcome are associated, even for relatively complex models (Friedman, 2001). In the case of models fitted using many covariates, it is possible to create a matrix of PDP for the most relevant features (Fig. 4) However, PDP presume the independence of features, meaning that feature(s) for which the partial dependence is computed are considered as not correlated with other features (Molnar, 2020).

In order to show the interaction of two interacting covariates, a surrogate model can be used. Surrogate models are interpretable models that approximate the black-box model up to a certain degree of accuracy. In this study Generalized linear mixed models (GLMMs) were used as surrogate models. GLMM are a class of models that incorporates random effects into the linear predictor of a generalized linear model, allowing the modelling of correlated data within the context of GLM (Gałecki and Burzykowski, 2013).

GLMM were used in this study to give an interpretation of the relation between Hg concentrations and the interaction of organic carbon content and NDVI quantiles (Fig. 5) and the interaction of organic carbon and the distance from coal power plants quantiles (Fig. 7).

## 3 Results

### 3.1 Hg pool in European topsoil

The deep learning model built to estimate topsoil Hg distribution in Europe was fitted against observations of topsoil Hg concentrations (LUCAS topsoil database) by gradient descent. Gradient descent is an iterative optimization algorithm for finding the local minimum of a function (see Fig. S1 in the Supplement). Variables were selected by regularization. In a first step, the DNN acted as an autoencoder selecting outliers. These outliers were removed from the main dataset, but were subject to further analysis (see Sect. 3.5) The fitted model performed well (r^2^ = 0.8, *p* < 0.01). K-fold cross-validation was used to avoid overfitting. The initial performance metrics is based on the root-mean squared error, however given the relatively high influence of higher values prediction, the mean absolute error (MAE) was used instead. Residuals from the DNN regression showed presence of spatial correlation. Thus, ordinary block kriging was used for interpolation residuals. The resulting map showing the spatial distribution of Hg residuals in the European topsoil was added to the DNN outcome in a regression kriging fashion (Hengl et al., 2007).

The average ± SD topsoil Hg concentration of all LUCAS samples was 55.4 ± 4.9 μg kg^−1^ and ranges between 0.054 and 49,200 μg kg^−1^. The median Hg concentration was 23.3 μg kg^−1^, with an inter-quartile range of 35.7 μg kg^−1^ (1st quartile 13.5 μg kg^−1^, 3rd quartile 49.2 μg kg^−1^). The 99th percentile of 429 μg kg^−1^ indicates that the top 1% of the measured Hg concentrations are likely to be outliers corresponding to sites subject to geogenically enriched bedrock or topsoil Hg contamination. The highest concentration (49,000 μg kg^−1^ or 49 ppm) was sampled nearby the Almadén Hg mine.

**Fig. 2:**
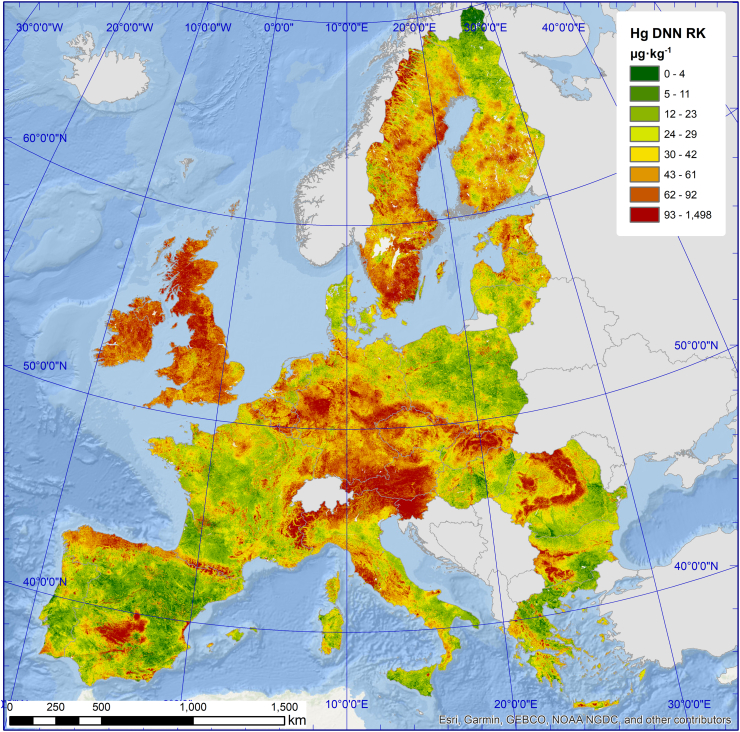
Topsoil Hg concentrations (μg kg^−1^) across 26 EU countries estimated by deep neural network – regression kriging. Colour classes are based on 12.5th percentiles.

**Fig. 3:**
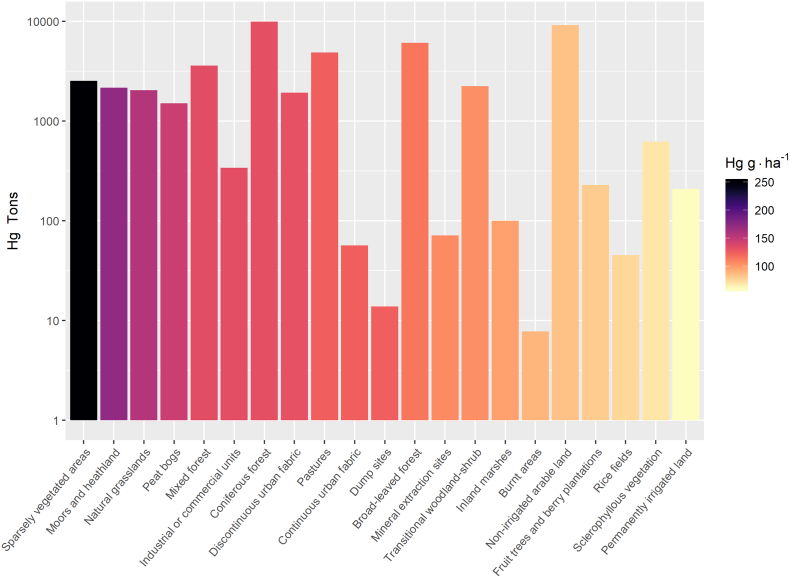
Soil Hg pool per land cover type (CORINE).

Land cover specific topsoil Hg concentrations (per hectare) and relative stocks of Hg are based on the DNN-regression kriging (Fig. 2) and CORINE, and are presented in Fig. 3. The average Hg concentration in natural grasslands is 70.4 μg kg^−1^ (0.13·10^6^ km^2^) Higher topsoil Hg concentrations in coniferous forests (56.4 μg kg^−1^, 0.75·10^6^ km^2^) relative to broad-leaved forests (49.7 μg kg^−1^, 0.53·10^6^ km^2^) have been previously described on smaller scale studies and were related to higher atmospheric Hg deposition fluxes in coniferous forests (Gruba et al., 2019; Richardson and Friedland, 2015). Agricultural land has generally lower average concentrations ranging from 33.4 μg kg^−1^ of non-irrigated arable land (1.09·10^6^ km^2^) to 23.1 μg kg^−1^ of permanently irrigated land (0.04·10^6^ km^2^) (Fig. 3). Taking into account the soil bulk density, the topsoil of EU contains approximately 44.8 Gg of Hg. This estimate accounts for the Hg concentration that can be predicted from the model taking into account larger scale variables such as geological background and factors driving atmospheric Hg deposition. The soil Hg pool is probably slightly underestimated as the survey might have missed localized contamination spots, which might constitute an additional portion of the Hg pool.

### 3.2 Influence of climate on topsoil Hg distribution

Climate appears to have a strong influence on the distribution of Hg on a continental scale. In cold climate regions such as high-latitude and high-altitude areas elevated soil Hg concentrations prevail (Fig. 2). The boreal zone of Sweden for example revealed average topsoil concentrations of 60 ± 53 μg kg^−1^. This observation is in the range of 10–115 μg kg^−1^ representing typical soil concentrations from boreal forest catchments (Skyllberg et al., 2003). Topsoil concentrations in Sweden but also in Ireland (78 ± 49 μg kg^−1^) or Great Britain (83 ± 65 μg kg^−1^) are elevated due to the large proportion of atmospheric Hg that is bound to soil organic matter (Jiskra et al., 2015; Meili, 1991; Obrist et al., 2011).

The north-south gradient in topsoil Hg concentrations from Sweden (60 ± 53 μg kg^−^) > Italy (49 ± 45 μg kg^−1^) is opposite to the gradient in Hg deposition by rain and snowfall from <5 μg m^−2^ A^−1^ in Scandinavia to >40 μg m^−2^ A^−1^ in parts of southern Europe (www.emep.int). Annual wet Hg deposition in Russia (<1 μg m^−2^ A^−1^) was an order of magnitude lower compared to wet deposition in Slovenia (Sprovieri et al., 2017). Increasing Hg concentrations with latitude have been reported for the United states (Obrist et al., 2011; Richardson et al., 2013) and western Siberia (Lim et al., 2020). In regions where woodland is the prevalent land cover type and humus-rich topsoil dominates (e.g. boreal zone) Hg concentrations are elevated despite lower inputs by wet atmospheric deposition.

Mountainous areas in Europe (above 600 m a. s. l.) show significantly higher (70 ± 62 μg kg^−1^) topsoil Hg concentrations compared to the lowlands (32 ± 29 μg kg^−1^) (*p* < 0.0001). This finding is consistent with field studies reporting an increase of atmospheric Hg deposition with altitude and larger Hg soil concentrations at higher elevations in the United States (Blackwell and Driscoll, 2015; Townsend et al., 2014) and China (Wang et al., 2017; Zhang et al., 2013). Exemplary, low topsoil Hg concentrations were found in Spain except for mountainous regions, e.g. the Pyrenees and the Cantabrian Mountains or the former Almadén Hg mining area southwest of the city of Madrid, a region where Hg-rich ore deposits exist (Millán et al., 2006).

**Fig. 4:**
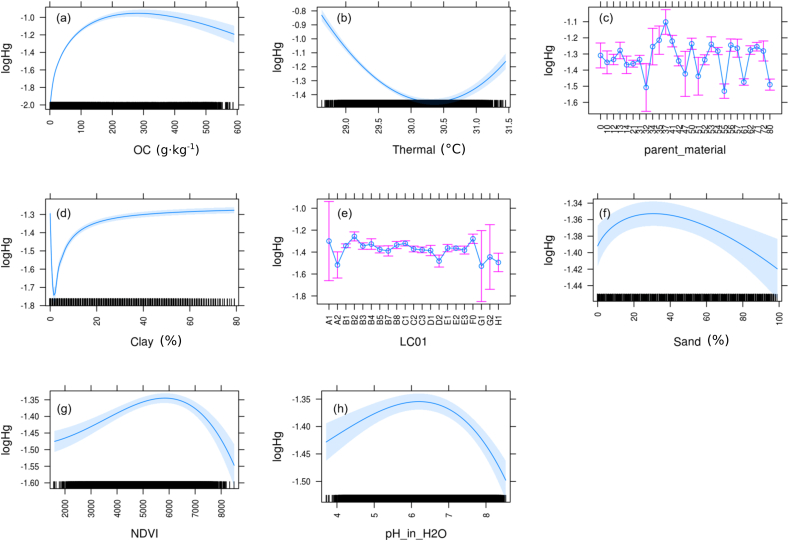
Partial dependency plots for the effect of the most influential variables on the model outcome. Parent material classes are reported in Table S1 and land cover classes (LC1) in Table 2.

The finding of decreasing topsoil Hg concentrations along the European north-south gradient is supported by the fact that overall Hg content decreases with increasing surface temperatures (Fig. 4b). The effect of surface temperature is represented by the thermal band of the Landsat satellites. Air and soil temperatures exhibit a strong positive relationship with gaseous elemental Hg (Hg^0^) air and surface temperatures (Edwards and Howard, 2013; Osterwalder et al., 2018; Zhang et al., 2001). Elevated temperatures reduce activation energy required to release Hg from soil or vegetation and subsequently increase Hg volatilisation rates to the atmosphere (Kim et al., 2012). Increasing temperatures likely enhance kinetically induced Hg(II) photo-reduction, producing Hg^0^ that evades to the atmosphere (Choi and Holsen, 2009; Gustin et al., 2002). Thus, elevated Hg^0^ emissions in warmer regions are likely to reduce topsoil Hg content. Hg in soils in the north of Europe are therefore more stable and less prone to Hg^0^ emission, resulting in larger overall stocks over the time of soil formation. An air temperature rise by 1–2 °C until 2050 is projected to increase Hg^0^ emissions from terrestrial surfaces globally by 15 to 43% (MacSween et al., 2019).

### 3.3 Influence of soil properties on topsoil Hg distribution

The increase of topsoil Hg concentrations with high latitudes and high elevation could at least partly be explained by an increase of organic carbon concentrations (Fig. 4a). Topsoil Hg concentrations increase with organic carbon content until a concentration of 200 g kg^−1^ is reached. Beyond this threshold, Hg concentrations decrease slightly. Such an increase of Hg concentration along with soil organic carbon has been described previously (Grigal, 2003; Obrist et al., 2011), and is likely related to the uptake of Hg^0^ by vegetation. The small decrease with carbon concentrations between 300 and 600 g kg^−1^ is likely due to the change in land cover or organic carbon type. Such high concentrations of organic carbon (typically >30%) are associated with peatlands (i.e. Histosols). Fast peat accumulation rates and inhibited re-mineralisation of carbon due to anoxic conditions may result in lower Hg pools stored in the top 20 cm of organic peat soils.

Our results indicate that soil Hg is not only bound to organic matter (Fig. 4a) but associated with iron oxide and clay minerals (Fig. 4d). Clay and iron oxides serve as sorption sites for metals such as Hg (Gabriel and Williamson, 2004; Skyllberg, 2010). The increase of topsoil Hg concentrations with clay content can be described with a logarithmic function. The function describing the correlation between Hg concentration and clay content reaches a plateau at 40% clay content. In contrast, topsoil Hg concentrations decrease with increasing sand content (beyond circa 30%) (Fig. 4f). Sand represents a relatively weak sorbent for Hg(II) (Sarkar et al., 1999). In sandy soils the abundance of high affinity Hg(II) sorbents, both clay and organic carbon, decreases making them less prone to Hg accumulation.

Furthermore, the relationship between topsoil Hg concentrations and soil solution pH indicates that Hg accumulation increases with pH, starting from a pH of 4 and reaching a maximum at a pH of 6.5 (Fig. 4h). Very low soil pH values are often related to anoxic wetlands where reducing conditions inhibit the re-mineralisation of carbon and favour the transformation of Hg(II) to Hg^0^. As, loss of soil Hg to the atmosphere through Hg^0^ evasion has been observed in anoxic wetlands (Fritsche et al., 2014; Osterwalder et al., 2017), very low pH soils (circa pH 4) are more likely to be associated with lower topsoil Hg concentrations.

Parent material also influences Hg content as the natural background concentrations of Hg varies among rock types (Fig. 4c). The parent material classes with the highest Hg content are found in mineralized regions characterized by subduction zones and volcanic deposits (Schlüter, 2000). However, parent material influence on soil chemical composition can vary due to factors like soil age, so that the topsoil can bear little relation (from a chemical point of view) to the parent material. The relation between Hg concentrations and parent material can be misleading in some cases and it can be further complicated by the geographical distribution of the substrate. For instance, loess deposits in Europe correspond with the areas of central Europe where Hg emissions are higher than average, so the effect of this substrate on Hg concentrations is likely due to its limited geographical extent more than to its chemical composition.

The effect of land cover is also evident with higher-than-average Hg concentrations in all the cropland classes (B) (Fig. 4e). However due to other factors (like a lower SOC content and higher average temperature), the final concentrations in cropland are in fact lower than those of other types of land cover as discussed in the following section.

### 3.4 Vegetation activity (NDVI/ C-content) as main driver of Hg topsoil distribution

**Fig. 5:**
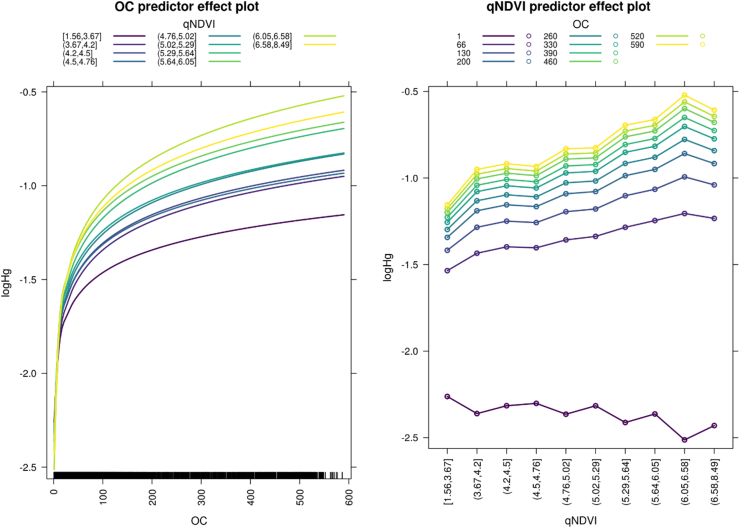
Effect plots of the GLMM showing the interaction between soil OC and NDVI and its influence on Hg distribution. The OC predictor effect plot (left) shows the relation between Hg concentrations and the concentration of soil OC and the lines colour depicts the value of the yearly cumulative NDVI subdivided in 10 quantiles. OC concentrations are in g.kg-1; NDVI yearly cumulative value is divided in 10 quantiles, with the numbers between parenthesis representing the interval of the quantile. The qNDVI predictor effect plot (right) depicts the change of Hg with the change of NDVI for different levels of SOC.

Jiskra et al. (2018) observed a covariation between atmospheric Hg^0^ concentrations and vegetation activity over the growing season. We tested if vegetation activity has also a control on the Hg pool in soil through the transfer of Hg via litterfall (Wang et al., 2016). As this rate of transfer is related to the amount of organic matter transferred from the canopy to the soil, higher amount of soil organic matter should correlate to both the NDVI and soil Hg concentrations. To test this hypothesis, a GLMM was fitted using Hg soil concentrations as the dependent variable and soil organic carbon plus NDVI quantiles as independent variables (Fig. 5). The model is relatively effective in predicting the spatial distribution of Hg across the EU (r^2^ = 0.35, *p* < 0.001). The model indicates that at least part of the Hg distribution is driven by the effect of vegetation. Obrist et al. (2016) observed a strong correlation of Hg concentrations in soils and organic carbon, precipitation, vegetation activity (NDVI) and foliar Hg mass in the western United States. The results are also in agreement with observations of a positive correlation between Hg concentrations in soils and both, Leaf area index (LAI), and SOC on the Tibetan plateau (Wang et al., 2016). However, we found no positive correlation between the lowest quantile of topsoil SOC (1 to 96 g kg^−1^) and NDVI. Since there are no deposition fluxes available, we hypothesise that in soils with low SOC, the sorption capacity is limited because of the fast mineralisation rate of OC (Fig. 5). When SOC concentrations are similar, soil Hg concentrations increase with NDVI (Fig. 5). The same effect is shown in the qNDVI (NVDI percentiles) predictor effect plot, where the increase Hg concentrations is related to the increase of the NDVI (represented by its quantiles), in this plot the colour represents the concentrations of OC in the soil.

The correlation of soil Hg concentrations in soils with qNDVI suggests that vegetation uptake of Hg^0^ and Hg transfer to soils is controlled by vegetation activity. This observation corroborates the importance of the vegetation Hg^0^ pump in the build-up of the soil Hg pool over continental scales. The slope of log_Hg_ as a function of qNDVI increases with increasing SOC indicating that the soil Hg pool becomes larger when high SOC concentrations prevail (Fig. 5). In summary, the results of the GLMM show an increasing Hg concentration in soils with increasing vegetation activity, represented by qNDVI, which is further amplified by higher SOC concentrations.

### 3.5 Soil Hg concentration hotspots related to mining and industrial activities

Approximately 3% of the points were classified as outliers by the autoencoder of the DNN and where removed by the regression model. However, while the distribution of these outliers cannot be easily explained by environmental features, their spatial distribution can provide useful information about their possible origin. By applying the trained DNN model on the full dataset of Hg measurements, we obtained residuals including the outliers. Residuals were tested for spatial correlation by building a robust variogram using Cressie's estimator. Then residuals were interpolated using block kriging (2 × 2 km block) in order to obtain a map of residuals including the outliers.

**Fig. 6:**
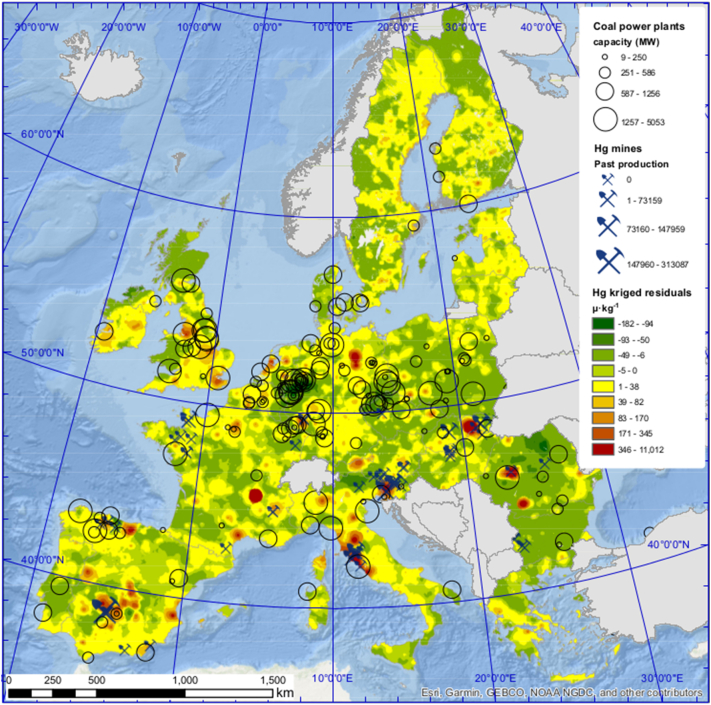
Map of kriged residuals including outliers. Overlaid are coal fired power plants in circles with the size of the circle proportional to the power production and Hg mines with the size of the symbols representing their past production (where available).

The resulting map is shown in Fig. 6 and illustrates that most soil Hg hotspots are located in central Europe and Great Britain, whereas no hotspots are present in Scandinavia. In total, we identified 209 hotspots, defined by a threshold of top 1 percentile of all measured values (>429 μg kg^−1^). We could associate 87 (42%) hotspots with known mining sites. The most important Hg mining sites in Europe, the Idrija mine in Slovenia (Gosar et al., 2016), the Almadén mine in Spain (Millán et al., 2006) and Monte Amiata (Rimondi et al., 2015) were identified in the residual map (Fig. 6). For the remaining 122 (58%) hotspots the source of Hg contamination could not be identified, despite the extensive set of variables tested. Unidentified Hg hotspots could be related to undocumented small-scale industrial pollution or geologically enriched Hg bedrock that has never been mined and is therefore not represented in the ProMine or the Minerals4EU databases.

**Fig. 7:**
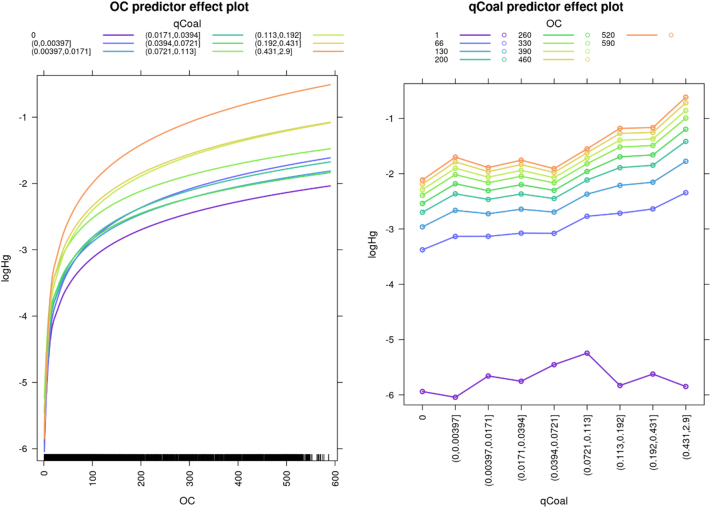
Effect plots of the GLMMl correlating Hg to the spatial density of the reported power production by coal power plants and soil OC. OC concentrations are in g kg^−1^; qCoal is the MW km^−2^ from coal estimated using a moving kernel of 10x10k divided into 10 quantiles, with the numbers between parenthesis representing the interval of the quantile.

Another source of Hg is represented by coal combustion in power plants in the EU (circles in Fig. 6)(Zhang et al., 2015). Hg emissions from coal fired power plants can be in the form of oxidized Hg^2+^ (approx. 65%) or as gaseous elemental Hg^0^ (approx. 35%), with the two species exhibiting different lifetimes in the atmosphere (Zhang et al., 2015, Zhang et al., 2016). Hg^2+^ emissions are deposited in proximity to the emission source, whereas Hg^0^ emissions are subject to global transport, with a lifetime of 8 to 13 months (Saiz-Lopez et al., 2018). Hg emissions from coal combustions can lead to elevated Hg levels in soils as shown for the largest power plant of Serbia (Dragović et al., 2013). The effect of coal power plant emissions to soil Hg levels can be evidenced by fitting a GLMM, correlating Hg concentrations, soil OC content and the spatial density of the coal power plants capacity (MWh km^−2^). There is a positive relationship between coal combustion and high levels of soil Hg (Fig. 7). This positive relationship is statistically significant for coal power plant capacities above 0.13 MWh km^−2^ (*p* < 0.002). We found that in areas with similar levels of soil OC, soil Hg concentrations were elevated when coal power plant capacities were large. Consequently, Hg emissions from coal fired power plants are associated with higher Hg deposition in the proximity of the plant, resulting in local soil Hg contamination. The installation of flue gas desulfuration to coal fired power plants led to a over-proportional decreases of Hg^2+^ to total emissions (Zhang et al., 2016) suggesting that the impact of coal power plants on local soil contamination was higher before flue gas desulfuration was installed in the EU. There is, however, no clear association of coal fired power plants with the Hg hotspots (top 1 percentile) with the exception of highly industrialized areas like e.g. Liverpool, London, or Milano-Bergamo (Fig. 6). The Hg contamination of soils in these metropolitan areas likely resulted from multiple pollution sources including coal combustion and metal smelting.

It is important to note that the coarse sampling resolution of LUCAS, one sample every ~200 km^2^, only captures anthropogenic point sources with a large radius or small pollution sites that lay on the sampling grid by chance. Nevertheless, several hotspots seem to be related to industrial pollution in particular in the areas surrounding active or formerly active metallurgy facilities. The source of Hg for other hotspots such as the ones in proximity of Lyon, Montpellier (FR), Berlin (DE), Bilbao and Valencia (ES) could not be identified in this study. The mapping effort in the framework of LUCAS can serve as a starting point to guide local and regional authorities in identifying Hg contamination hotspots in soils. Data and maps will be made available in the European Soil Data Centre (ESDAC) (Panagos et al., 2012).

## 4 Conclusions and future action

The current study is based on a much higher number of samples (21,591) compared to the 4329 samples of the GEMAS study (Ottesen et al., 2013); in addition, the LUCAS survey is based on stratified random sample (and not a regular grid) allowing for better estimates and mapping. Moreover, the LUCAS survey has the advantage of not being limited to agricultural and grassland soils, but samples a broader range of ecosystems (including forests and wetlands) thus providing a more detailed picture of the distribution of Hg across the European Union.

Moreover other studies (Reimann et al., 2018b) were limited to a purely spatial interpolation of the Hg concentrations or were based on a small set of covariates with a very low spatial resolution (5 km) (Lado et al., 2008). In addition, the study of Lado et al. (2008) was further limited by the lack of covariates characterizing soil properties (a critical driver of soil Hg concentrations) as by the lack of information about some of the potential sources of mercury (I.e. mining sites, power plants, etc.).

The LUCAS topsoil heavy metals database already proved to be able provide detail information allowing the assessment of other potential contaminants, as it was used to analyse the spatial distribution and the factors influencing copper distribution in EU soils (Ballabio et al., 2018).

Given the characteristics of the LUCAS survey, the broad range of covariates considered and the mapping technique, the present study provides the best map, in terms of resolution and accuracy, of the distribution of Hg in the topsoil of EU so far produced. Moreover, the use of DNN allowed the identification of outliers and the use of multiscale data. The analysis of the DNN output by GLMM, provides additional information about the drivers of topsoil Hg contamination and their interactions. Finally, we use the spatially continuous estimates provided by the DNN-kriging mapping along with soil data to provide reliable estimates of the stocks of mercury in the EU topsoil for different types of land cover.

There is a high variation of Hg concentration across the EU, mainly due to impact of natural processes (high soil organic carbon, vegetation, parent material, temperature, soil texture and pH) and some evident high values close to past mining activities and coal combustion sites. High concentrations of mercury have been found close to well-known mining sites like Almaden (Asturias, Spain), Mt. Amiata (Italy), Idrija (Slovenia) and Rudnany (Slovakia). In a more detailed investigation, 42% of the hotspots were associated with well-known mining activities while the rest can be related either to coal combustion industries or local diffuse contamination.

Overall, the stock of Hg in EU topsoil is estimated to c.a. 44.8 Gg with a median concentration of 38.3 μg kg^−1^; 10% of the area exceeds the 84.7 μg kg^−1^ and 209 Hg hotspots (top 1%) have been identified with concentrations >422 μg kg^−1^.

According to our study, diffuse Hg contamination in European topsoil is not an emerging issue. However, policy actions are needed for managing the existing hotspots and the emissions connected to power production.

## Credit author statement

**Cristiano Ballabio**: Conceptualization, Methodology, Software, visualization, Writing original draft and revision, Formal Analysis, Validation, Investigation.

**Martin Jiskra**: Conceptualization, Writing original draft, Investigation.

**Stefan Osterwalder**: Conceptualization, Writing original draft, Investigation.

**Pasquale Borrelli**: Data Curation, Writing original draft, Investigation.

**Luca Montanarella**: Supervision.

**Panos Panagos**: Conceptualization, Writing original draft, Supervision, Investigation.

## Declaration of competing interest

The authors declare that they have no known competing financial interests or personal relationships that could have appeared to influence the work reported in this paper.

## Appendix A Supplementary data

Supplementary material Image 1
