# Supplementary material for: A spatial assessment of mercury content in the European Union topsoil
Source: Sci Total Environ. 2021 May 15;769:144755. doi: 10.1016/j.scitotenv.2020.144755 (PMC8024745; doi:10.1016/j.scitotenv.2020.144755)
Supplement: Supplementary file 1 — Supplementary material [file mmc1.docx]

# Supplementary Material

**An assessment of mercury content in EU soils**

Cristiano Ballabio^1^*, Martin Jiskra^2^, Stefan Osterwalder^3^, Luca Montanarella^1^, Panos Panagos^1^

^1^European Commission, Joint Research Centre (JRC), Ispra (VA), Italy.

^2^Environmental Geosciences, University of Basel, Basel, Switzerland.

^3^Université Grenoble Alpes, CNRS, IRD, Grenoble INP, IGE, Grenoble, France

Corresponding author:

*E-mail: Cristiano.ballabio@ec.europa.eu

Contents:

S1. Parent material type and related codes from the European Soil Database p. 2

S2. Geostatistical Analysis of LUCAS Hg topsoil concentrations p. 2

S3. Variable importance deep learning p. 3

**S1: Parent material type and related codes from the European Soil Database**

| Tab. S1: Parent material type and codes as derived from the European Soil Database. | |  |
| --- | --- | --- |
| **Parent material type** | **Code** | |
| no information | **0** | |
| consolidated-clastic-sedimentary rocks | **10** | |
| psammite or arenite | **12** | |
| pelite, lutite or argilite | **13** | |
| facies bound rock | **14** | |
| calcareous rocks | **21** | |
| acid to intermediate plutonic rocks | **31** | |
| basic plutonic rocks | **32** | |
| acid to intermediate volcanic rocks | **34** | |
| basic to ultrabasic volcanic rocks | **35** | |
| pyroclastic rocks | **37** | |
| metamorphic rocks | **41** | |
| acid regional metamorphic rocks | **42** | |
| tectogenetic metamorphism rocks | **47** | |
| unconsolidated deposits | **50** | |
| marine and estuarine sands | **51** | |
| marine and estuarine clays and silts | **52** | |
| fluvial sands and gravels | **53** | |
| fluvial clays, silts and loams | **54** | |
| lake deposits | **55** | |
| residual and redeposited loams from silicate rocks | **56** | |
| residual and redeposited clays from calcareous rocks | **57** | |
| morainic deposits | **61** | |
| glaciofluvial deposits | **62** | |
| loess | **71** | |
| eolian sands | **72** | |
| organic materials | **80** | |

**S2: Geostatistical Analysis of LUCAS Hg topsoil concentrations**

| **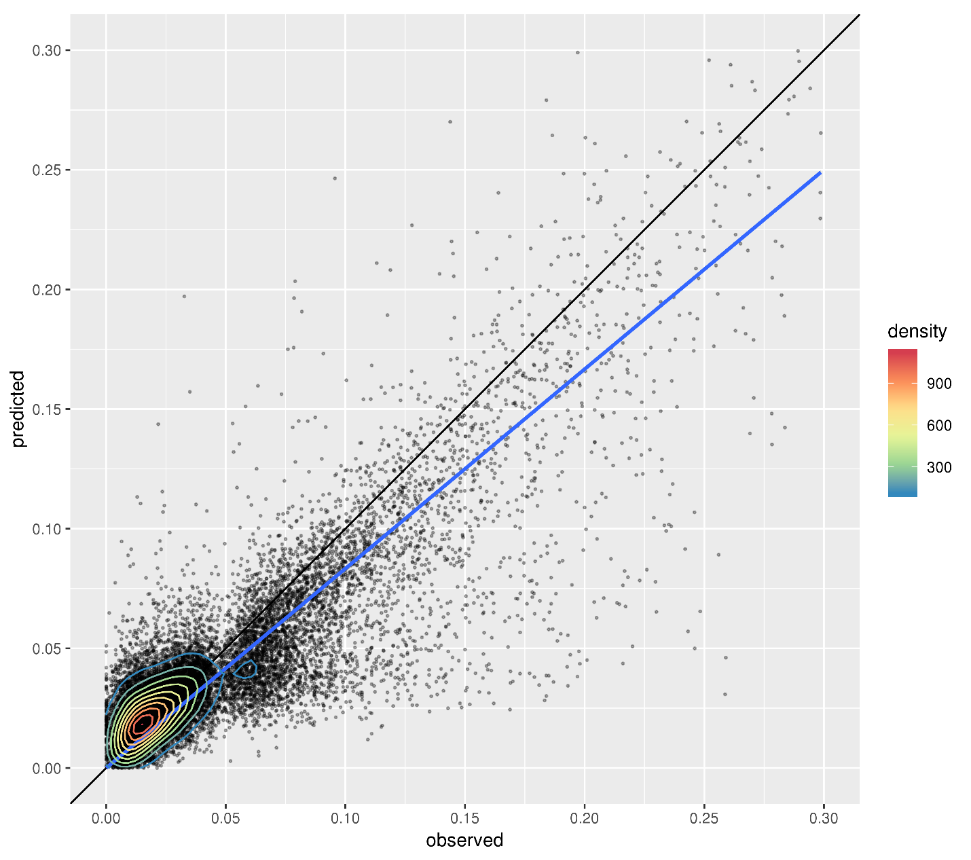** |
| --- |
| **Figure S1:** Plot of predicted vs measured Hg concentrations. The blue line shows the linear regression fitting and the black line the diagonal. Density isolines are overlaid to show the point density, evidencing that the vast majority of the observations falls in |

| 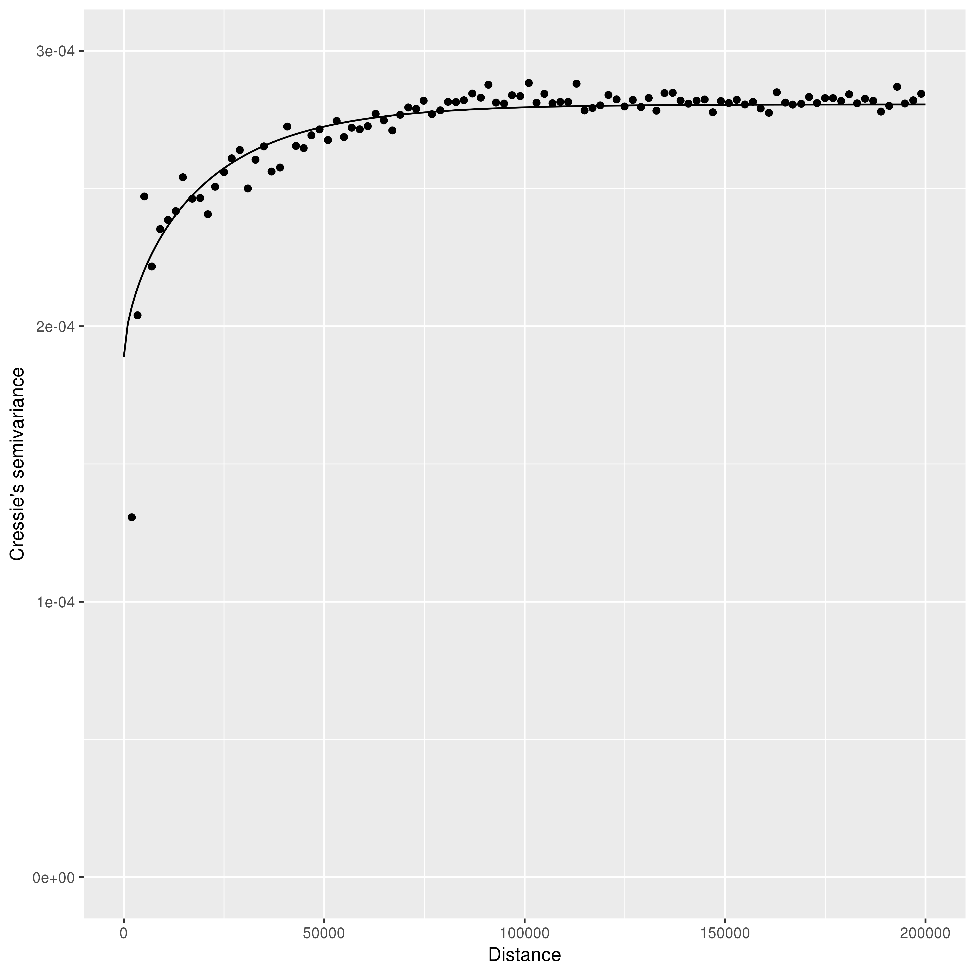 |
| --- |
| **Figure S2:** Variogram of the DNN residuals computed using Cressie robust semivariance. |

| 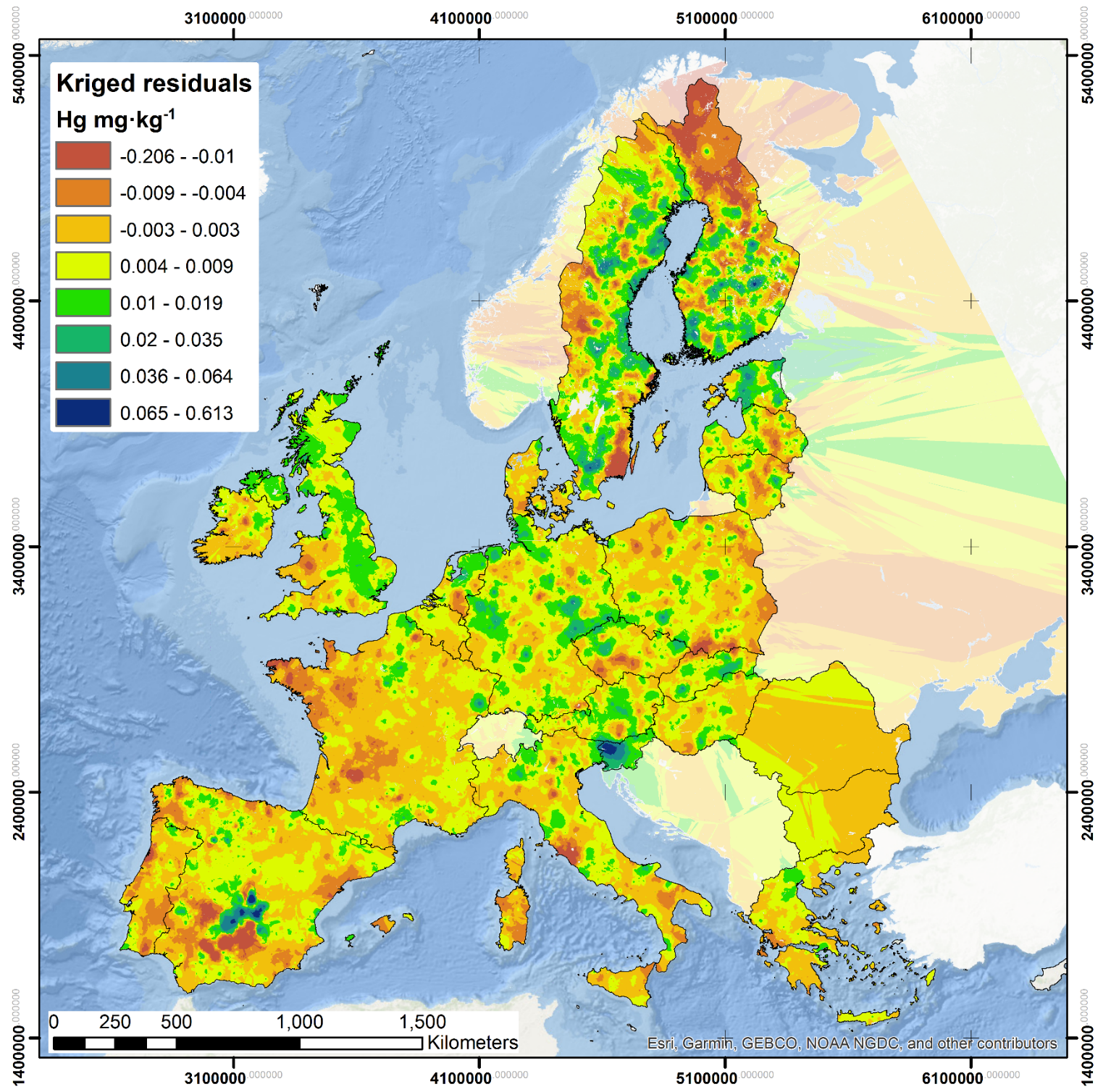 |
| --- |
| **Figure S3:** Interpolated residuals |

**S3: Variable importance deep learning**

| **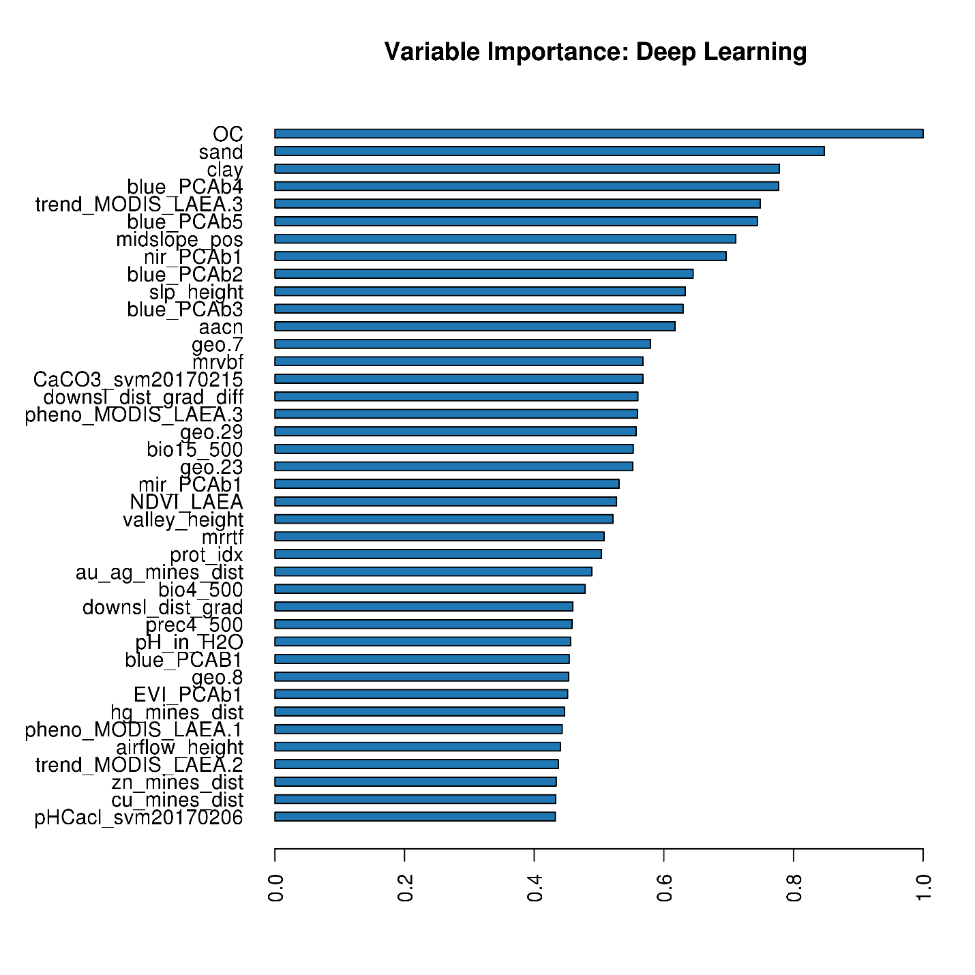** | |
| --- | --- |
| **Figure S4:** Variable importance for the exploratory trained model. This model includes a mixture of variables, including soil properties as measured on field samples, and proxies. The model evidences the high relevance of soil properties influencing the absorption and retention of Hg, mostly driven by Organic Carbon and soil texture. OC: Organic Carbon; sand: sand content; clay: clay content; blue_PCAb4: 4th component of the PCA rotation of the year 2009 16days MODIS blue band; midslope_pos: topographic midslope position; trend_MODIS_LAEA.3: trend of the yearly MODIS NDVI; nir_PCAb1: 1st component of the PCA rotation of the year 2009 16days MODIS near infrared band; slp_height: vertical elevation of slope; aacn: altitude above channel network; geo.7:… ; mrvbf: multiresolution valley bottom flatness index; CaCO3_svm20170215: soil calcium carbonates content; downs_dist_grad_diff: downslope distance gradient difference; pheno_MODIS_LAEA.3: phenological cycle component of the MODIS NDVI; geo.29 … ; NDVI_LAEA: Averaged yearly NDVI |  |

| **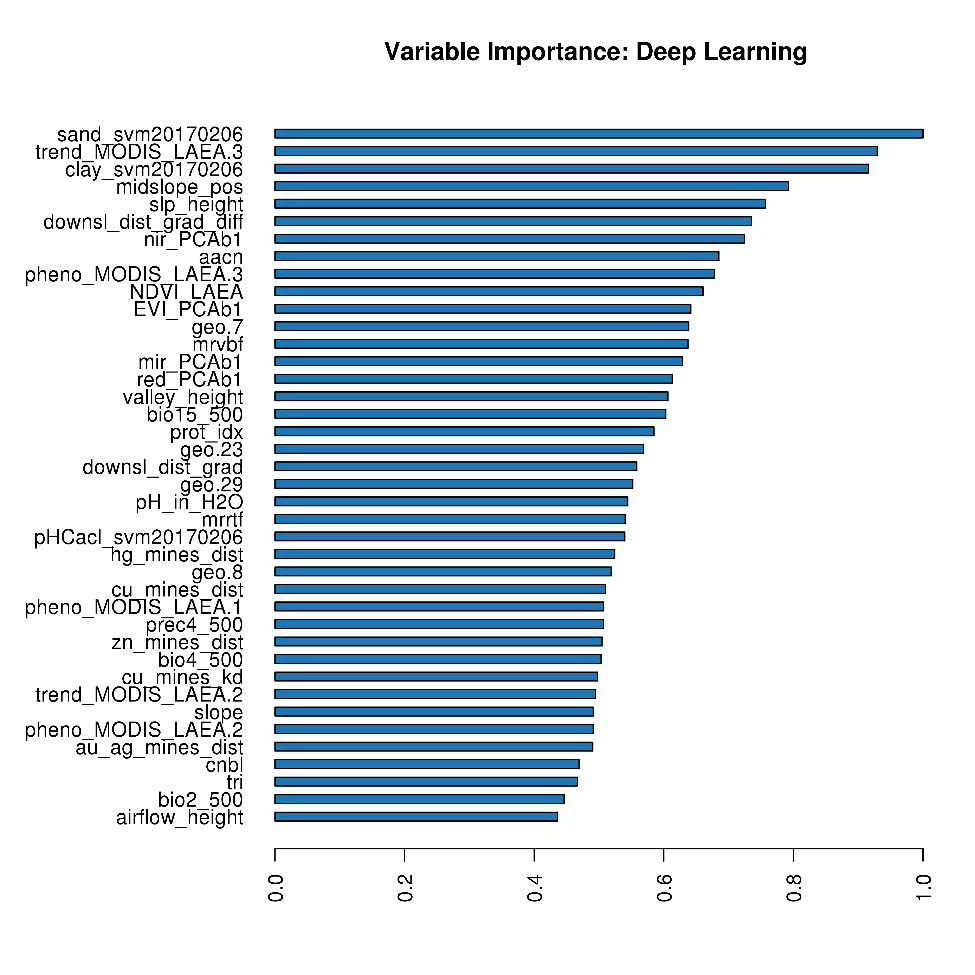** | |
| --- | --- |
| **Figure S5:** Variable importance for the mapping trained model. This model includes spatially exhaustive proxies and was used in the mapping stage. |  |
